# Neonatal Zika virus infection causes transient perineuronal net degradation

**DOI:** 10.3389/fncel.2023.1187425

**Published:** 2023-07-11

**Authors:** Kaliroi Engel, Ha-Na Lee, Bhanu P. Tewari, Aaron P. Lewkowicz, Derek D. C. Ireland, Mohanraj Manangeeswaran, Daniela Verthelyi

**Affiliations:** ^1^Laboratory of Immunology, Center of Excellence in Infectious Disease and Inflammation, Office of Biotechnology Products, Center for Drug Evaluation and Research, Food and Drug Administration, Silver Spring, MD, United States; ^2^Department of Neuroscience, School of Medicine, University of Virginia, Charlottesville, VA, United States

**Keywords:** Zika virus (ZIKV), brain development, perineuronal net (PNN), Wisteria floribunda agglutinin (WFA), aggrecan, brevican, metalloproteinase (MMP)

## Abstract

Perineuronal nets (PNNs) form a specialized extracellular matrix that predominantly surrounds parvalbumin (PV)-expressing GABAergic inhibitory interneurons and help regulate neuronal activity. Their formation early in the postnatal period is regulated by neuronal signaling and glial activation raising concerns that part of the long-term effects ascribed to perinatal viral infections could be mediated by altered PNN formation. Previously, we developed a model of neonatal Zika virus (ZIKV) infection where mice have lifelong neurological sequelae that includes motor disfunction and reduced anxiety coupled with a persistent low-grade expression in proinflammatory markers despite resolving the acute infection. Here, we demonstrate that ZIKV infection to P1 neonatal mice results in a reduction of PNN formation during the acute disease with significant reduction in Wisteria floribunda agglutinin (WFA) staining at the peak of infection [15 days post infection (dpi)] that persisted after the symptoms resolved (30 dpi). At 60 dpi, when there is residual inflammation in the CNS, the number of WFA^+^ cells and the level of WFA staining as well as levels of aggrecan and brevican in the brains of convalescent mice were not different from those in uninfected controls, however, there was increased frequency of PNNs with an immature phenotype. Over time the impact of the perinatal infection became less evident and there were no clear differences in PNN morphology between the groups at 1 year post infection. Of note, the reduction in PNNs during acute ZIKV infection was not associated with decreased mRNA levels of aggrecan or brevican, but increased levels of degraded aggrecan and brevican indicating increased PNN degradation. These changes were associated with increased expression of matrix metalloproteinase 12 (MMP12) and MMP19, but not MMP9, a disintegrin and metalloproteinase with thrombospondin motifs 4 (ADAMTS4) or ADAMTS5. Together our findings indicate that infection at the time of PNN development interferes with PNN formation, but the nets can reform once the infection and inflammation subside.

## Introduction

Perineuronal nets (PNNs) are a specialized extracellular matrix surrounding mostly parvalbumin (PV)-positive GABAergic inhibitory neurons in various brain regions including the cortex, hippocampus, amygdala, hypothalamus, basal ganglia, and cerebellum, although some non-PV neurons produce them as well (Shi et al., 2019; Wingert and Sorg, 2021). The lattice-like PNNs are composed of hyaluronic acid, tenascin-R, and the lectican family of chondroitin sulfate proteoglycans (CSPGs) (e.g., aggrecan, brevican, neurocan, and versican) (Bandtlow and Zimmermann, 2000; Ueno et al., 2018a). Their formation is known to increase with neuronal activity and brain maturation and can be modulated by microglial activity (Dityatev et al., 2007; Tewari et al., 2022). Indeed, these dynamic mesh-like structures around neurons are thought to be critical for neuronal plasticity and memory by stabilizing synapses and acting as physical barriers for electrical impulses (Bruckner et al., 2006; Sorg et al., 2016; Shi et al., 2019). This includes PNNs in the hippocampus and the anterior cingulate cortex function which protect long-term fear memory by regulating feedback inhibition of parvalbumin interneurons (Shi et al., 2019). Supporting these functional roles, histochemical loss of PNN matrices has been reported for a range of neurocognitive and neurodegenerative diseases such as Alzheimer’s disease (AD), epilepsy, and schizophrenia, indicating that PNNs are likely key players in the pathogenesis of neurological disorders. Less well understood is whether perinatal stress can modulate the development of these PNNs and how that impacts neurocognitive capabilities long-term.

Disruption of PNN formation has been reported in mouse models of neurodegenerative disorders such as AD, Creutzfeldt–Jakob disease and schizophrenia as well as acute viral encephalitis (Bissel et al., 2012; Bitanihirwe and Woo, 2014). Growing evidence suggests that upregulation of matrix metalloproteinase 9 (MMP-9) activity during central nervous system (CNS) inflammation promotes proteolytic degradation of PNN components such as aggrecan and brevican, resulting in PNN impairment (Reinhard et al., 2015). The earliest evidence of PNN formation in mouse brains is at postnatal day 3 (P3) in the reticular nuclei, followed by in the primary sensory cortices and other nuclei of the brain stem by P7 (Horii-Hayashi et al., 2015), but formation and maturation of PNNs evolve at different rates in different areas of the brain (Horii-Hayashi et al., 2015). At this time, the impact of perinatal infections on PNN formation including the potential role of glial activation in PNN formation remains unclear.

To investigate this, we used a mouse neonatal Zika virus (ZIKV) infection model previously described, where challenging P1 neonatal mice (C57BL/6J) with ZIKV (1000 TCID_50_ of ZIKV PRVABC59, subcutaneously) leads to a non-lethal meningoencephalitis (Manangeeswaran et al., 2016). In this model, live virus is consistently present in the brain at day 6 and peaks at 9–12 days post-infection (dpi); the infection is associated with reduced weight gain, ataxia, widened gait, kinetic tremors, and seizures that peak at 15 dpi but resolve 1–2 weeks later. By 30 dpi, viral titers and inflammation marker expression in the brain as well as behavioral changes are markedly reduced. Interestingly, while only 10–20% of the animals succumb to infection and survivors have a normal life-span, imaging showed increased levels of fluid in CNS and reduced cerebellum volume and behavioral studies showed lifelong abnormalities including impaired motor coordination and reduced endurance as well as reduced anxiety in the surviving mice (Ireland et al., 2020). Using this neonatal ZIKV infection model, here we examined whether acute ZIKV infection at the time of PNN development affects PNN formation by analyzing the intensity and the area of Wisteria floribunda agglutinin (WFA) staining and the degradation of aggrecan and brevican in the brains of ZIKV-infected mice at 15, 30, and 60 dpi.

## Materials and methods

### Mice

C57BL/6J (B6) mice were purchased from Jackson Laboratory (JAX; Bar Harbor, ME). Mice were bred and housed in the specific pathogen-free, AAALAC accredited animal facility of the U.S. Food and Drug Administration’s Division of Veterinary Medicine (Silver Spring, MD). Mice were housed in standard cages with 1 breeding pair or up to 5 single sex mice per cage and a 12/12 light/dark schedule and fed on commercial 5P76 Prolab Isopro RMH 3000 diet. The experimental protocol 2019-02 was reviewed and approved by the FDA Animal Care and Use Committee (FDA-ACUC), and all animals used in these studies conform to relevant regulatory standards. All procedures were performed in accordance with the FDA ACUC guidelines.

### ZIKV infection

ZIKV PARVABC59 (Puerto Rico strain), isolated by the CDC [explained in the previous publication (Manangeeswaran et al., 2016; Ireland et al., 2020)], was used for this study. One-day-old (P1) neonatal mice were inoculated with 1000 TCID_50_ (50% tissue culture infectious dose) of ZIKV by subcutaneous injection. All procedures were performed as described (Manangeeswaran et al., 2016; Ireland et al., 2020) and in accordance with the FDA-ACUC guidelines.

### Brain immunohistochemistry and confocal imaging

Mice were euthanized by CO_2_ asphyxiation and exsanguinated by intracardiac perfusion with 10mL of PBS. Brain tissues were collected and bisected sagittally. One hemisphere was snap-frozen in liquid nitrogen and kept at −70°C for Western blot analysis. The other hemisphere of the brain from each mouse was collected and immediately placed in 4% paraformaldehyde for 24 h before being transferred into 30% (w/v) sucrose solution until the brains were fully infused. Brains were then embedded in TissueTek O.C.T (Sakura-Finetek, Torrance, CA) mounting media and 30 μm sections cut using a Leica CM1900 cytostat (Leica Biosystems, Buffalo Grove, IL), mounted onto Superfrost Plus microscope slides (Fisher Scientific, Hampton, NH) and stored at −80°C. For immunofluorescence-immunohistochemistry (IF-IHC), slides were warmed and allowed to dry at room temperature. The sections were then hydrated in 1 × PBS (Gibco, Waltham, MA), followed by antigen retrieval in sodium citrate buffer, pH 9.0 at 80°C for 8 min and 10 min at room temperature (RT). The sections were then permeabilized in Triton X-100 for 1 h at RT and incubated for at least 1 h at RT in 1 × PBS containing 5% normal goat serum, 1% bovine serum albumin and 0.05% triton X-100 to block non-specific binding. The blocked sections were stained with biotinylated wisteria floribunda lectin (WFA, WFL) (Vector Laboratories, Newark, CA) for 24 h at RT. After washing, sections were then incubated with Alexa-fluor conjugated streptavidin (MilliporeSigma, Burlington, MA) in dilution buffer for at least 2 h at RT. All slides were mounted with Prolong Diamond Anti-Fade mounting media containing DAPI (ThermoFisher, Carlsbad, CA). Whole section images were acquired using an Olympus VS-120 Virtual Microscope, using Olympus VS software (Olympus LSS). The somatosensory cortex and the CA2 region of the hippocampus were imaged using a Zeiss LSM 880 confocal microscope and the Zeiss Zen software (Carl Zeiss Inc., Oberkochen, Germany). Maximum intensity projections of acquired Z-stacks were compiled using Zeiss Zen and ImageJ software (NIH, MD).

### WFA quantification

Multichannel 3D images were converted into hyperstacks of individual channels using ImageJ software, and WFA intensity was analyzed as previously described (Schindelin et al., 2012). Briefly, max intensity z projections of the red channel were created and used for analysis. ROIs were drawn to determine background intensity and measurement. Threshold intensity was determined by directly measuring the field intensity and area of the images after background subtraction.

### Protein extraction and western blot analysis

Proteins were extracted by grinding half brains in the radioimmunoprecipitation assay (RIPA) buffer [20 mM Tris–HCl (pH 7.5), 150 mM NaCl, 1 mM Na_2_EDTA, 1 mM EGTA, 1% Triton X-100, 2.5 mM sodium pyrophosphate, 1 mM β-glycerophosphate, 1 mM Na_3_VO_4_, 1 mg/ml leuptin, 1 mM phenylmethanesulphonyl- fluoride (PMSF)] containing protease and phosphatase inhibitors for 1 h on ice, followed by centrifugation for 15 min at 12,000 × *g*. Protein lysates (15 μg) were electrophoresed by sodium dodecyl sulfate (SDS)-polyacrylamide gel electrophoresis (PAGE) and then separated proteins were transferred to polyvinyl difluoride (PVDF) membrane (Millipore Sigma, MA). To block the non-specific binding of proteins with primary antibodies, the blots were incubated in 5% non-fat dry milk-TBST buffer [tris-buffered saline containing 0.1% Tween-20] for 1 h at room temperature. The membranes were then incubated with rabbit anti-aggrecan (Millipore Sigma, Burlington, MA) and mouse anti-brevican (BioLegend, San Diego, CA) antibodies suspended in 3% non-fat milk TBST buffer overnight at 4°C. This was followed by washing with TBST and incubation using appropriate secondary antibody coupled to horseradish peroxidase. Proteins tagged with specific primary antibodies were visualized with an enhanced chemiluminescence detection kit (ThermoFisher). The band intensity was measured using Gel-Pro analyzer 3.0 (Media Cybernetics, Rockville, MD).

### Real-time PCR

Total RNA was extracted from brain tissues using Trizol (Invitrogen, Waltham, MA). After DNase treatment (DNA-free Turbo kit; ThermoFisher), 1 μg of total RNA was used for cDNA synthesis using Multiscript High Capacity Reverse Transcriptase (ThermoFisher) using random primers per manufacturer’s protocol. Gene expression assays were performed using a Viia7 real-time PCR machine (ThermoFisher) with QuantStudio software (ThermoFisher). Fold changes in gene expression were determined by normalizing Ct values to GAPDH (housekeeping gene) and then to age-matched uninfected controls.

### Statistical analysis

Statistical significances were determined by the 2-tailed unpaired Student’s *t*-test using GraphPad Prism (version 9.1.2; GraphPad Software, CA). *P* < 0.05 was considered statistically significant.

## Results

### Neonatal ZIKV infection causes PNN degradation during early PNN development stages

**FIGURE 1:**
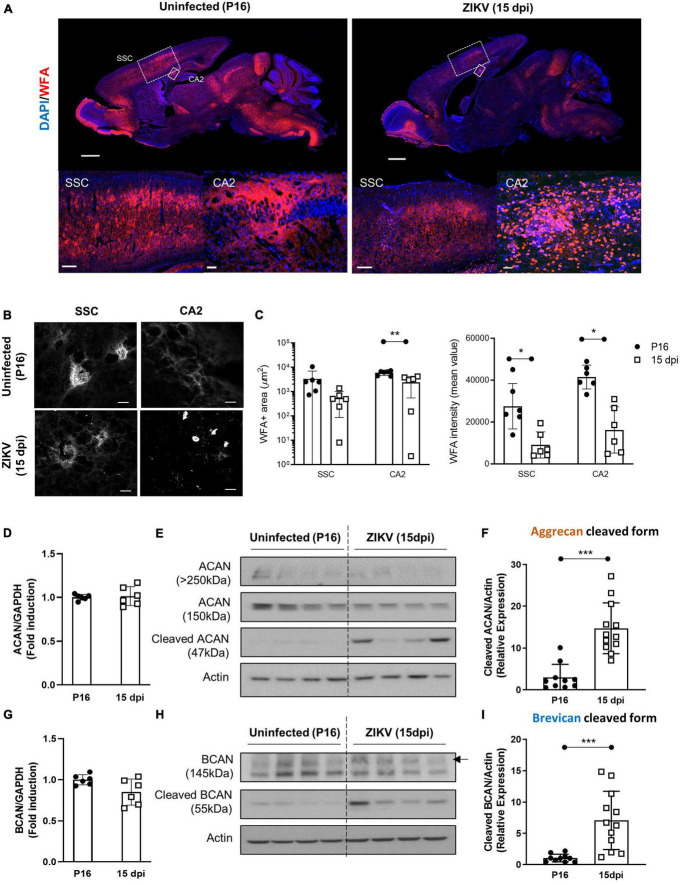
Impact of ZIKV infection on early PNN development. PNN formation was assessed in the brain of P16 mice uninfected or infected with 1000 TCID_50_ of ZIKV on P1. **(A)** Top: representative immunofluorescence staining for WFA (red) and DAPI (blue) in the sagittal brain sections. Scale bars: 1 mm. Bottom: higher power images show representative immunofluorescence staining for WFA in SSC and CA2 regions (scale bars: 200 and 40 μm, respectively). *N* = 3 for each group. **(B)** Confocal imaging of PNNs in SSC and CA2 regions of uninfected and infected mice. *N* = 3 for each group. Scale bars: 20 μm. **(C)** Quantitation of WFA^+^ area (number of fluorescent pixels) and the WFA intensity (mean value of fluorescent pixels). Data shown as means ± S.D. (*n* = 1–2 sections per brain from 3 mice in each group). *P < 0.05, **P < 0.01 (student’s *t*-test). **(D,G)** Relative mRNA levels of aggrecan (ACAN) **(D)** and brevican (BCAN) **(G)** in the brain from uninfected (P16) and ZIKV-infected mice at 15 dpi. Data shown as means ± S.D. (*n* = 6 mice for each group). **(E,H)** The protein levels of aggrecan **(E)** and brevican **(H)** in brain tissue lysates as detected by Western blot analysis. Full-length aggrecan bands at >250 kDa and the cleaved forms bands at 150 and 47 kDa **(E)**. Full-length brevican band at 145 kDa and the cleaved forms bands at 55 kDa **(H)**. Actin band was used for loading normalization. Western blots show four representative samples per group. **(F,I)** Quantitation of cleaved aggrecan and brevican bands. Data shown as means ± S.D. (*n* = 10–12 mice for each group). ***P < 0.001 (student’s *t*-test).

In previous studies, we showed that a subcutaneous challenge of P1 C57BL/6 mice with 1000 TCID_50_ of ZIKV leads to CNS infection, which peaks at 9 dpi and then diminishes over the following weeks (Manangeeswaran et al., 2016). This was accompanied by increased inflammation in the brain starting by 3 dpi and peaking at 15 dpi, as evidenced by the expression of immune-related genes (Ireland et al., 2020). Given that the period of acute infection coincides with that of active PNN formation (Horii-Hayashi et al., 2015; Supplementary Figure 1A), we used this model to examine whether the viral infection and subsequent inflammation at a critical neurodevelopmental age would alter PNN formation. To examine PNN formation, we assessed the staining with Wisteria floribunda agglutinin (WFA), a lectin that selectively labels the N-acetylgalactosamine residue on chondroitin sulfate (CS) chains in PNNs (Mirzadeh et al., 2019), in the brains of uninfected (P16) and ZIKV-infected mice (15 dpi). Since at P15-P16 a significant amount of chondroitin sulfate proteoglycans (CSPG) is present in diffused form, we examined overall levels of CSPG using WFA staining. As shown in Figure 1A, there was an overall reduction in WFA staining throughout the brain in infected mice. While PNNs were evident in several regions of the brain, we chose to focus on somatosensory cortex (SSC) and CA2 region of the hippocampus because the explicit emergence of PNNs allows for quantification and the CA2 region of the hippocampus is associated with memory (Chevaleyre and Piskorowski, 2016). In addition, our previous study has shown that while these areas are frequently infected by the virus, they do not cause overt structural changes/destruction to the tissue architecture (Manangeeswaran et al., 2016). Both regions show reduced mean WFA intensity and area as compared to age-matched uninfected mice (Figures 1B, C). At the single cell level, PNNs in the uninfected SSC can be observed as condensed coats on the cell soma as well as around the proximal processes (Figure 1B), however in the infected SSC, PNNs coating around the cell soma appeared highly diffused and nearly absent around the neuronal processes (Figure 1B). Of note, the infected brains showed smaller round shaped cells strongly stained with WFA, however, they were identified not as PNN but as a subset of CD45^+^ infiltrating cells (Poretz et al., 1986; Supplementary Figure 2). Next, we investigated whether the reduction in WFA staining in the brain of ZIKV-infected mice was secondary to the absence of aggrecan and brevican, which are key components of PNNs (Sonntag et al., 2018). Interestingly, ZIKV infection did not alter mRNA expression or total protein levels of aggrecan and brevican, however, it led to an increase in the cleaved forms of aggrecan and brevican proteins (Figures 1D–I). These findings suggest that neonatal ZIKV infection results in PNN component degradation rather than reduced formation of PNN components.

### Reduced PNN formation during the infection resolution phase

**FIGURE 2:**
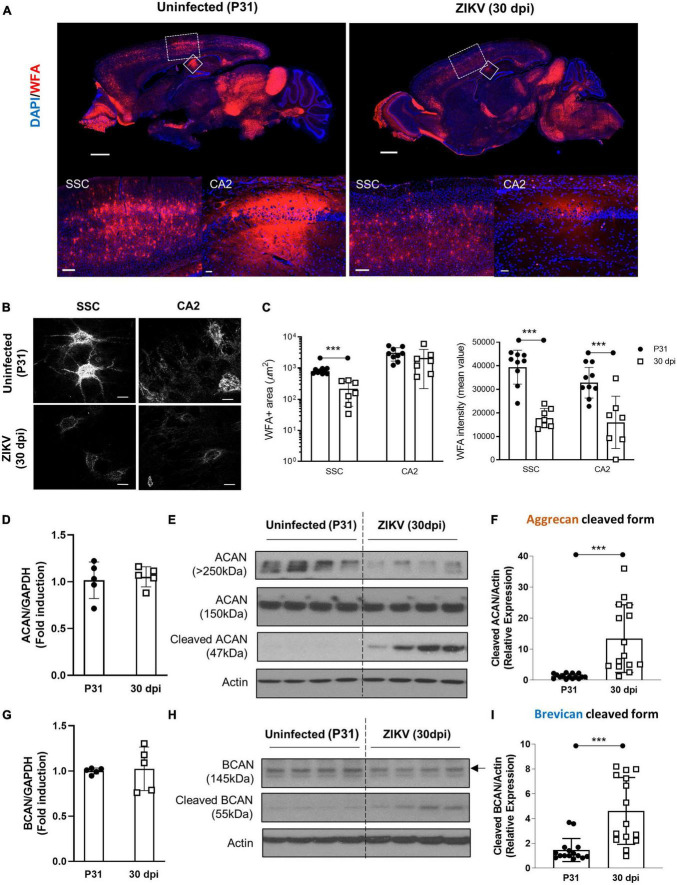
A loss of PNN formation in the brain of ZIKV-infected mice at the resolution phase. PNN formation was assessed in the brain of P31 mice uninfected or infected with 1000 TCID_50_ of ZIKV on P1. **(A)** Top: representative immunofluorescence staining for WFA (red) and DAPI (blue) in the sagittal brain sections. Scale bars: 1 mm. Bottom: higher power images show representative immunofluorescence staining for WFA in SSC and CA2 regions (scale bars: 200 and 40 μm, respectively). *n* = 3 for each group. **(B)** Confocal imaging of PNNs in SSC and CA2 regions of uninfected and infected mice. *n* = 3 for each group. Scale bars: 20 μm. **(C)** Quantitation of WFA^+^ area (number of fluorescent pixels) and the WFA intensity (mean value of fluorescent pixels). Data shown as means ± S.D. (*n* = 2–3 sections per brain from 3 mice in each group). ***P < 0.001 (student’s *t*-test) **(D,G)** Relative mRNA levels of ACAN **(D)** and BCAN **(G)** in the brain from uninfected (P31) and ZIKV-infected mice at 30 dpi. Data shown as means ± S.D. (*n* = 5 mice for each group). **(E,H)** The protein levels of aggrecan **(E)** and brevican **(H)** in brain tissue lysates as detected by Western blot analysis. Actin band was used for loading normalization. Western blots show four representative samples per group. **(F,I)** Quantitation of cleaved aggrecan and brevican bands. Data shown as means ± S.D. (*n* = 15 mice for each group). ***P < 0.001 (student’s *t*-test).

After the peak of infection at 15 dpi, ZIKV-infected animals that survive show reduced symptoms, inflammation and viral load in their brain (Manangeeswaran et al., 2016; Ireland et al., 2020; Supplementary Figures 1A, B). To determine whether PNN formation is restored following the resolution of the acute phase of ZIKV infection, we quantified WFA staining in the brain of ZIKV-infected mice at 30 dpi as compared to age-matched controls. As shown in Figures 2A–C, the brains of ZIKV-infected mice showed less WFA staining including in the SSC and CA2 regions compared to uninfected mice suggesting reduced PNN levels in infected mice compared to uninfected controls. Since the PNNs were now fully formed in uninfected mice, the difference in PNNs structural integrity at the single PNN level was much more evident. At 30 dpi, PNNs in the uninfected brain showed highly condensed and extensive WFA coating on soma, primary and secondary dendrites and the axonal initial segment (AIS), however, PNNs in the infected brain still appeared highly diffused on cell soma and failed to show any arborized condensation (Figure 2B). As observed at 15 dpi, despite no differences in mRNA levels of aggrecan and brevican (Figures 2D, G), the increase in cleaved aggrecan and brevican remained significantly higher in the brain of infected mice indicating a persistent impact of the infection on the integrity of the proteoglycans (Figures 2E, F, H, I).

### PNN restoration post-infection

**FIGURE 3:**
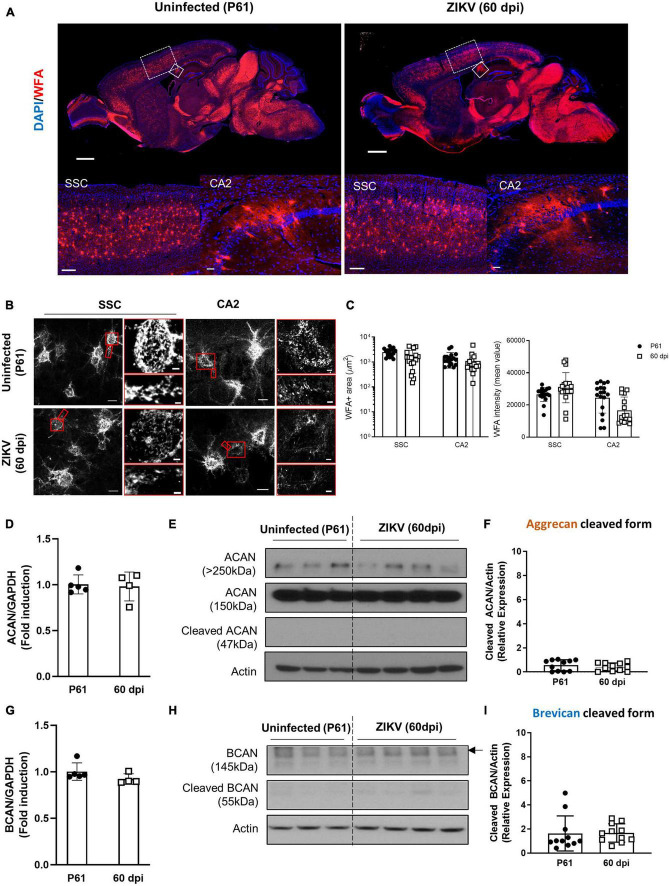
Perineuronal net restoration in convalescent mice. PNN formation was assessed in the brain of P61 mice uninfected or infected with 1000 TCID_50_ of ZIKV on P1. **(A)** Top: representative immunofluorescence staining for WFA (red) and DAPI (blue) in the sagittal brain sections. Scale bars: 1 mm. Bottom: higher power images show representative immunofluorescence staining for WFA in SSC and CA2 regions (scale bars: 200 and 40 μm, respectively). *n* = 6 for each group. **(B)** Confocal imaging of PNNs in SSC and CA2 regions of uninfected and infected mice. Scale bars: 20 μm. Increased magnification highlighting PNN morphology in SSC and CA2 regions (red box, scale bar: 2 μm). *n* = 6 for each group. Scale bars: 1 mm **(C)** Quantitation of WFA^+^ area (number of fluorescent pixels) and the WFA intensity (mean value of fluorescent pixels). Data shown as means ± S.D. (*n* = 2–3 sections per brain from 3 mice in each group). **(D,G)** Relative mRNA levels of ACAN **(D)** and BCAN **(G)** in the brain from uninfected (P61) and ZIKV-infected mice at 60 dpi. Data shown as means ± S.D. (*n* = 4–5 mice for each group). **(E,H)** The protein levels of aggrecan **(E)** and brevican **(H)** in brain tissue lysates as detected by Western blot analysis. Actin band was used for loading normalization. Western blots show four representative samples per group. **(F,I)** Quantitation of cleaved aggrecan and brevican bands. Data shown as means ± S.D. (*n* = 11 mice for each group).

**FIGURE 4:**
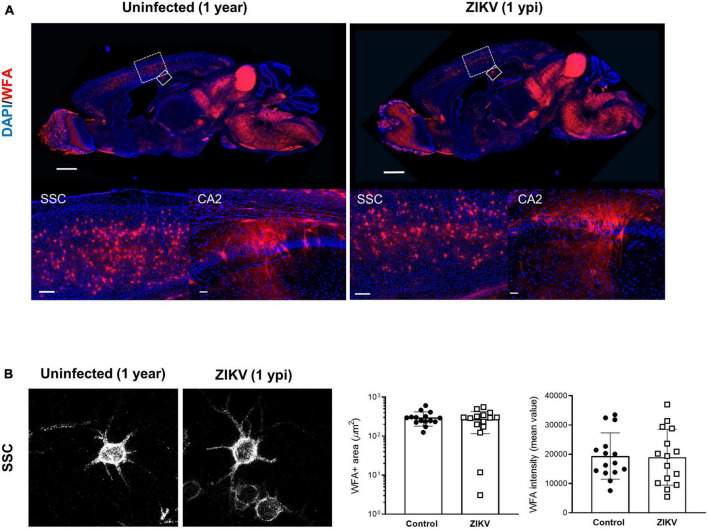
WFA levels in convalescent mice at 1 year post infection. One year old mice uninfected or infected with 1000 TCID_50_ of ZIKV on P1. **(A)** Top: representative immunofluorescence staining for WFA (red) and DAPI (blue) in the sagittal brain sections. Scale bars: 1 mm. Bottom: higher power images show representative immunofluorescence staining for WFA in SSC and CA2 regions (scale bars: 200 and 40 μm, respectively). *n* = 3 for each group. **(B)** Confocal imaging of PNNs in SSC regions of uninfected and infected mice at 1 year post infection. Quantitation of WFA^+^ area (number of fluorescent pixels) and the WFA intensity (mean value of fluorescent pixels). Data shown as means ± S.D. (*n* = 5 sections from 3 mice in each group).

Previous work showed that by 60 dpi, ZIKV-infected mice have only vestigial viral infection in isolated foci of the cerebellum and markers of inflammation return to baseline (Ireland et al., 2020; Supplementary Figures 1A, B). To determine whether the PNN formation can be restored after the infection subsides, we assessed the WFA levels in 60 dpi mice. As shown in Figures 3A–C, by 60 dpi there was no significant difference in the WFA staining intensity between control and convalescent mice, and similar levels of PNN formation were detectable surrounding the soma of neurons in the SSC and the hippocampus. Further, in addition to no differences in the mRNA levels for aggrecan and brevican, the levels of cleaved forms of aggrecan and brevican were indistinguishable between convalescent mice and the age-matched controls (Figures 3D–I). However, careful examination of the images suggested a disruption in PNN morphology, with areas where WFA staining appears more diffuse, disconnected, less condensed and less arborized, suggesting an immature PNN phenotype at 60 dpi (Horii-Hayashi et al., 2015; Sigal et al., 2019), as compared to the age-matched mice (Figure 3B and Supplementary Figure 3A). Interestingly, a year after infection there were no differences in WFA staining or PNN morphology between control and convalescent mice (Figure 4 and Supplementary Figure 3B), suggesting that PNNs can form later in development and dysregulation of PNN maturation is not persistent. These findings are consistent with the previously reported dynamic nature of the PNN formation and suggest that the overall level of PNN formation is fully restored in convalescent mice.

### MMP-12 and -19 could play a role in PNN degradation during ZIKV infection

**FIGURE 5:**
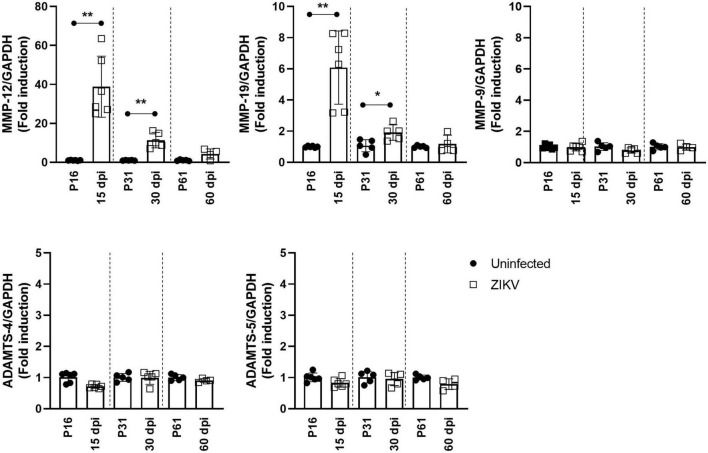
mRNA expression of MMPs and ADAMTSs in the brain of ZIKV-infected mice. Relative mRNA levels of MMP-12, MMP-19, MMP-9, ADAMTS-4, and ADAMTS-5 in the brain of uninfected or ZIKV-infected mice at 15, 30, and 60 dpi were determined using real-time RT PCR. Data shown as means ± S.D. (*n* = 4–6 mice for each group). *P < 0.05, **P < 0.01.

ZIKV infection is characterized by astrocyte and microglial activation as well as monocyte infiltration (Manangeeswaran et al., 2016). The proinflammatory factors released by these cells are thought to modulate PNN formation and stability (Crapser et al., 2021; Tewari et al., 2022). Factors identified as playing a key role in PNN degradation include MMPs and a disintegrin and metalloproteinase with thrombospondin motifs (ADAMTS) (Tewari et al., 2018). Given that ZIKV infection induces proteoglycan degradation, we next examined whether the infection was associated with increased expression of MMPs and ADAMTSs. As shown in Figure 5, increased mRNA levels for MMP-12 and, to a lesser extent, MMP-19 were observed in the brain of ZIKV-infected mice at 15 and 30 dpi when there is increased degradation of aggrecan and brevican and reduced PNN levels (Figures 1, 2), but their levels had returned to baseline at the time of PNN restoration (Figure 3) and reduced inflammation (Supplementary Figure 1B). On the other hand, mRNA levels for MMP-9, ADAMTS-4, and ADAMTS-5, which have been reported associated with PNN degradation, and gelatinase/collagenase activity were not significantly elevated in the brain during ZIKV infection (Figure 5 and Supplementary Figure 4). Together, these data suggest that the expression of MMP-12 and -19 could be associated with PNN degradation during ZIKV infection.

## Discussion

Perineuronal nets are a specialized form of extracellular matrix that surround the soma and proximal dendrites of PV-positive interneurons in several brain regions including the cortex and hippocampus. They control neuroplasticity and synaptogenesis and protect PV and other fast spiking neurons from oxidative stress (Seeger et al., 1994; Sorg et al., 2016; Ueno et al., 2018b). They also limit lateral mobility of molecules on the neuronal membrane and regulate access to some growth factors (van’t Spijker and Kwok, 2017). PNNs undergo a gradual and orderly development until achieving fully condensed arborization during prenatal and early postnatal development when synapses mature and stabilize (Mauney et al., 2013; Horii-Hayashi et al., 2015; Chaunsali et al., 2021). PNN components are derived from neurons and glia and their development is tied to neuronal signaling, as sensory deprivation within the critical period was shown to disrupt the normal development of PNNs and brain circuits, as well as glial activation (Chaunsali et al., 2021). There is extensive data associating tissue damage with PNN disruption and neurodegenerative diseases such as AD and adult-onset epilepsies (Bitanihirwe and Woo, 2014; Chaunsali et al., 2021), however, the impact of perinatal neurotropic viral infections on PNN development had not been explored. In this study, using a model of ZIKV neonatal infection, we showed that the development of PNNs is disrupted by infection resulting in a marked reduction in PNN density that is associated with an increase in cleaved forms of aggrecan and brevican. Interestingly, although the infection disrupted a well-established chronology of PNN development, once the infection and inflammation subsided the level of cleaved proteoglycans as well as the overall WFA staining distribution and PNN morphology resembled that of uninfected controls suggesting that PNNs can form and mature at a later time in development. Of note, the disruption in PNN formation during infection was associated with increased expression of MMP-12 and -19 but not MMP-9 or ADAMTS-4 or -5, which have been previously shown to be increased in some disorders where established PNNs are disrupted (Roughley and Mort, 2014; Carceller et al., 2020).

Brain plasticity is maximal early in development, when sensory experience drives neuronal migration, circuit formation and synaptic pruning (Kolb and Gibb, 2011). The development of PNNs appears to play a key role in this process, and maturation of PNNs is thought to be critical to limiting the formation of new synapses (Cisneros-Franco et al., 2020). This allows for sequential consolidation of connections and retention of new and more complex perceptual, motor, and cognitive functions (Cisneros-Franco et al., 2020). Consistent with this, the timing of PNN formation is progressive and specific for different brain regions (Horii-Hayashi et al., 2015). For example, WFA staining of the CA1 hippocampal region in the mouse brain becomes evident around P14 and matures by P21, whereas in the amygdala they become evident by P21 (Horii-Hayashi et al., 2015). Visualization of PNNs using WFA shows mature PNNs as bright lattice-like condensation predominantly juxtaposing the soma, dendrites, and axon initial segment (Slaker et al., 2016; Carstens et al., 2021). As shown in Figures 1, 2, infection of the CNS results in dampening of WFA staining with disorganization around neurons or dendritic arbors. The reduction was associated with an increase in the levels of degraded aggrecan and brevican. Interestingly, once the infectious process subsided (60 dpi), PNNs formed around the soma, dendrites and AIS suggesting that the formation of PNNs could be deferred in time, but initially were relatively less organized than those of aged-matched controls. In addition, there was a relative increase in “ghost PNNs” (Sigal et al., 2019; Venturino et al., 2021) in the SSC region as compared to uninfected controls (Supplementary Figure 3A), suggesting that loss of neurons may have occurred after the PNNs were established. Importantly, in adult mice, there was no significant difference in PNN frequency or morphology between groups, suggesting that the long-term behavioral and motor deficits observed in the mice (Ireland et al., 2020) are more likely a result of structural sequelae from the virus and not necessarily due to sustained abnormal PNN patterns. Of note, while our studies indicate that the total levels of PNNs are restored and the morphology of the SSC PNNs becomes indistinguishable from control mice over time, future, more detailed studies will need to investigate whether the restoration of PNNs extends to other areas of the CNS and whether the delayed formation of PNNs follows the original pattern of development, mirrors the progression of the infection/resolution, or occurs synchronously throughout the brain as the inflammation is controlled. In addition, the impact of the delay on synaptogenesis and connectivity will need to be explored in more detail.

Few studies have looked at the impact of viral encephalitis on PNN stability, although a reduction in PNN frequency has been described in the brains of subjects with HIV-associated neurocognitive disorders (HAND) (Bozzelli et al., 2020). This is possibly because most murine models of viral meningoencephalitis are lethal. Indeed, to our knowledge this is the first study to model the impact of early post-natal non-lethal viral encephalitis, which are common in early childhood, on PNN formation. Our results suggest that the CNS of ZIKV-infected mice has reduced WFA staining intensity, and to a lesser extent, reduced WFA^+^ area, suggesting a reduction in organized PNNs that evolves with the disease, being minimal at 15 dpi when the CNS has high viral titers and CNS inflammation, but restoring after the local inflammation resolves. The ability to restore PNNs after the stress has subsided is in agreement with previous studies showing that PNNs can re-form following spinal injury (Lemons et al., 2001) or chemical disruption (Bruckner et al., 1998). However, other studies have suggested that a proinflammatory maternal milieu, such as that induced by poly I:C inoculation, can associate with disruption of PNNs later in life (Paylor et al., 2016). In our model, we have previously shown that neonatal ZIKV infection leads to a reduced motor coordination and a hyperactive phenotype with reduced anxiety and thigmotactic tendencies, but no changes in social interaction or memory (Ireland et al., 2020). The motor changes correlated with brain fluid content and cerebellum volume, however it is not possible, at this time, to determine whether the delayed PNN formation contributes to the long term behavioral, or motor changes observed.

Changes in PNN frequency have been described in several neurodevelopmental and psychiatric disorders and, these differences have been replicated in animal models. For example, a few studies show that administration of Poly I:C during pregnancy, a strategy often used to model schizophrenia, results in reduced aggrecan staining in the hippocampus (Wegrzyn et al., 2021; Mao et al., 2022), and in a similar model, Paylor et al. (2016) described a reduction in WFA staining in the prefrontal cortex of the adult offspring. Similarly, altered PNN levels have been observed in murine models of Alzheimer’s disease that replicate the PNN loss observed in autopsies of AD patients (Crapser et al., 2020). In those models, the activation of microglia appears to mediate the PNN reduction (Tewari et al., 2022). Given the microglial activation induced by the virus and the temporal progression of the PNN development it is tempting to speculate that activated microglia, TNF, and/or other inflammatory cytokines produced by the glia are critical to the reduced formation of PNNs during the infection, however, our attempts to demonstrate this by using mice lacking TNF or by reducing microglia in the CNS resulted in more severe disease, so it has not been possible to directly demonstrate their role in the delay of PNN formation during infection.

Aggrecan is a member of the lectican family of proteoglycans, a family that includes versican, brevican, and neurocan (Miyata and Kitagawa, 2016). Some studies have suggested the possibility that aggrecan molecules regulate plasticity during postnatal development (Ueno et al., 2018b). Previous studies show that aggrecan is expressed late in embryonic development (∼E14) and its level increases steadily until 5 months in rats (Milev et al., 1998; Matthews et al., 2002). Brevican shows a highly similar developmental pattern to aggrecan (Milev et al., 1998). However, little is known about the post-translational mechanism for aggrecan and brevican or their stability and turnover. In our study, although WFA staining intensity was dramatically reduced, the levels of total aggrecan and brevican as detected by western blot, remained fairly constant, suggesting that neurons are capable of producing them despite the inflammatory environment. During acute infection and while the proinflammatory cytokines were elevated, the staining intensity of WFA remained low and the cleaved forms of aggrecan and brevican were elevated suggesting increased degradation during infection and inflammation. Interestingly, and possibly enabled by the maintenance of elevated aggrecan and brevican levels through adulthood, a restoration of PNN fluorescent intensity coincided with reduced aggrecan and brevican cleavage. Assuming that both changes are related and given that only a fraction of aggrecan and brevican was cleaved, it seems more likely that the reduced formation of PNNs exposed proteoglycans to degradation, rather than to speculate that the cleavage of aggrecan and brevican interfered with PNN development. Further studies exploring the dynamics of formation and degradation of PNN may help elucidate this.

Metalloproteinases including MMPs and ADAMTSs control the structure and function of the extracellular matrix (ECM) and PNNs in the brain. Changes in their activation levels have been associated with nervous system development and synaptic plasticity as well as with neurological diseases and neurodegeneration following stroke and spinal cord injury (Muri et al., 2019). In the ZIKV-infected mice, we found increased levels of MMP-12 and -19, while other proteases more frequently associated with PNN degradation were not. MMP-12 has previously been associated with blood-brain barrier disruption via tight junction protein degradation after focal cerebral ischemia (Chelluboina et al., 2015), while MMP-19 was associated with gliomas (Luo et al., 2018), but no studies had associated them with PNN formation or degradation. Disparate findings of elevated MMPs and ADAMTSs in PNN are not uncommon as systematic assessments of these proteases are lacking. For example, increased levels of MMP-2 and -9 have been associated with BBB failure in multiple sclerosis, while MMP-3 and -13 were increased in the brains of subjects with HAND (Bozzelli et al., 2020). On the other hand, multiple different MMPs including MMP-2, -3, -8, -9, and -13 have been associated with BBB degradation (Muri et al., 2019). A more exhaustive regional characterization of MMPs and ADAMTSs may provide additional clues as to their involvement in the regulation of PNN formation in newborns.

In summary, our studies show that perinatal ZIKV infection can lead to a postponement of the formation of PNNs. The reduced formation of PNNs was associated with an increase in MMP-12 and -19 expression and PNN proteolysis, both of which subside once the viral load and corresponding inflammation resolve. Neonatal mice infected with ZIKV show long term sequelae that are characterized by motor, coordination and behavioral consequences. The role of delayed PNN formation in the long-term consequences of neonatal infections remains unknown and needs to be elucidated.

## Data availability statement

The original contributions presented in this study are included in this article/Supplementary material. Uncropped gels are included in Supplementary Figure 5. Further inquiries can be directed to the corresponding author.

## Ethics statement

The animal study was reviewed and approved by the FDA Animal Care and Use Committee (FDA-ACUC).

## Author contributions

KE, HNL, and DV designed the study. KE and HNL mainly performed the experiments, analyzed the data, and wrote the manuscript. BT analyzed the confocal images. AL and DI helped with the experiments. HNL, BT, MM, and DV discussed the data. DV revised the manuscript. All authors contributed to the article and approved the submitted version.

## Supplementary material

Supplementary Figure 1 The disease and inflammation progression during ZIKV infection. **(A)** Schematic diagram depicting the disease progression of ZIKV infection. P1 C57BL/6 mice infected with ZIKV show the CNS infection that peaks at 9–15 dpi, together with a transient meningeal encephalitis characterized by unsteady gait, kinetic tremors, ataxia and seizures that appear 10–12 dpi and subside by 26–30 dpi. ZIKV-infected mice can show behavioral sequelae as late as 60 dpi. **(B)** Relative mRNA levels of TNF, IL-1β, IL-6, and IFNβ were determined in the brain of uninfected or ZIKV-infected mice at 15, 30, and 60 dpi using real-time RT PCR. Data shown as means ± S.D. (*n* = 4–6 mice for each group). **P* < 0.05, ^**^*P* < 0.01 (student’s *t*-test).

Supplementary Figure 2 Wisteria floribunda agglutinin (WFA) staining is colocalized with CD45 staining in round shaped cells observed in ZIKV-infected brains. Confocal imaging of WFA and CD45 staining in the CA2 regions of the brain from uninfected or ZIKV-infected mice at P16/15 dpi and P31/30 dpi. The images show representative immunofluorescence staining for DAPI (blue), WFA (red) and CD45 (green) in brain sections.

Supplementary Figure 3 Perineuronal net morphology in control and convalescent mice. Two representative confocal images (63×) show immunofluorescence staining for WFA in SSC regions of age-matched controls and convalescent mice at 60 dpi **(A)** and 1 ypi **(B)**. Blue circles, ghost PNNs; red lines, PNN on dendritic arbor; green arrows, PNN on AIS.

Supplementary Figure 4 Gelatinase/collagenase activity in the brain of ZIKV-infected mice. Gelatinase/collagenase activity was measured in the brain lysates from uninfected and ZIKV-infected mice at the indicated time point. PC, positive control (0.1 U/mL of *Clostridium* collagenase) is red bar. *n* = 2–3 mice for each group.

Supplementary Figure 5 Uncropped gel images. Full length gel images of aggrecan **(A)** and brevican **(B)** corresponding to the cropped images shown in Figures 1E, H, 2E, H, 3E, H.

## References

[B1] Bandtlow C. E. Zimmermann D. R. (2000). Proteoglycans in the developing brain: New conceptual insights for old proteins. *Physiol. Rev.* 80 1267–1290. 10.1152/physrev.2000.80.4.1267 11015614

[B2] Bissel S. J. Giles B. M. Wang G. Olevian D. C. Ross T. M. Wiley C. A. (2012). Acute murine H5N1 influenza A encephalitis. *Brain Pathol.* 22 150–158. 21714828 10.1111/j.1750-3639.2011.00514.x PMC3204170

[B3] Bitanihirwe B. K. Woo T. U. (2014). Perineuronal nets and schizophrenia: The importance of neuronal coatings. *Neurosci. Biobehav. Rev.* 45 85–99. 10.1016/j.neubiorev.2014.03.018 24709070 PMC4447499

[B4] Bozzelli P. L. Caccavano A. Avdoshina V. Mocchetti I. Wu J. Y. Conant K. (2020). Increased matrix metalloproteinase levels and perineuronal net proteolysis in the HIV-infected brain; relevance to altered neuronal population dynamics. *Exp. Neurol.* 323:113077. 10.1016/j.expneurol.2019.113077 31678140 PMC7919751

[B5] Bruckner G. Bringmann A. Hartig W. Koppe G. Delpech B. Brauer K. (1998). Acute and long-lasting changes in extracellular-matrix chondroitin-sulphate proteoglycans induced by injection of chondroitinase ABC in the adult rat brain. *Exp. Brain Res.* 121 300–310. 10.1007/s002210050463 9746136

[B6] Bruckner G. Szeoke S. Pavlica S. Grosche J. Kacza J. (2006). Axon initial segment ensheathed by extracellular matrix in perineuronal nets. *Neuroscience* 138 365–375. 10.1016/j.neuroscience.2005.11.068 16427210

[B7] Carceller H. Guirado R. Ripolles-Campos E. Teruel-Marti V. Nacher J. (2020). Perineuronal nets regulate the inhibitory perisomatic input onto parvalbumin interneurons and gamma activity in the prefrontal cortex. *J. Neurosci.* 40 5008–5018. 10.1523/JNEUROSCI.0291-20.2020 32457072 PMC7314408

[B8] Carstens K. E. Lustberg D. J. Shaughnessy E. K. Mccann K. E. Alexander G. M. Dudek S. M. (2021). Perineuronal net degradation rescues CA2 plasticity in a mouse model of Rett syndrome. *J. Clin. Invest.* 131:e137221. 10.1172/JCI137221 34228646 PMC8363283

[B9] Chaunsali L. Tewari B. P. Sontheimer H. (2021). Perineuronal net dynamics in the pathophysiology of epilepsy. *Epilepsy Curr.* 21 273–281. 10.1177/15357597211018688 34690566 PMC8512927

[B10] Chelluboina B. Klopfenstein J. D. Pinson D. M. Wang D. Z. Vemuganti R. Veeravalli K. K. (2015). Matrix metalloproteinase-12 induces blood-brain barrier damage after focal cerebral ischemia. *Stroke* 46 3523–3531. 10.1161/STROKEAHA.115.011031 26534974

[B11] Chevaleyre V. Piskorowski R. A. (2016). Hippocampal area CA2: An overlooked but promising therapeutic target. *Trends Mol. Med.* 22 645–655. 10.1016/j.molmed.2016.06.007 27372610

[B12] Cisneros-Franco J. M. Voss P. Thomas M. E. De Villers-Sidani E. (2020). Critical periods of brain development. *Handb. Clin. Neurol.* 173 75–88. 32958196 10.1016/B978-0-444-64150-2.00009-5

[B13] Crapser J. D. Arreola M. A. Tsourmas K. I. Green K. N. (2021). Microglia as hackers of the matrix: Sculpting synapses and the extracellular space. *Cell. Mol. Immunol.* 18 2472–2488. 10.1038/s41423-021-00751-3 34413489 PMC8546068

[B14] Crapser J. D. Spangenberg E. E. Barahona R. A. Arreola M. A. Hohsfield L. A. Green K. N. (2020). Microglia facilitate loss of perineuronal nets in the Alzheimer’s disease brain. *EBioMedicine* 58:102919. 10.1016/j.ebiom.2020.102919 32745992 PMC7399129

[B15] Dityatev A. Bruckner G. Dityateva G. Grosche J. Kleene R. Schachner M. (2007). Activity-dependent formation and functions of chondroitin sulfate-rich extracellular matrix of perineuronal nets. *Dev. Neurobiol.* 67 570–588. 10.1002/dneu.20361 17443809

[B16] Horii-Hayashi N. Sasagawa T. Matsunaga W. Nishi M. (2015). Development and structural variety of the chondroitin sulfate proteoglycans-contained extracellular matrix in the mouse brain. *Neural Plast.* 2015:256389. 10.1155/2015/256389 26649203 PMC4663360

[B17] Ireland D. D. C. Manangeeswaran M. Lewkowicz A. P. Engel K. Clark S. M. Laniyan A. (2020). Long-term persistence of infectious Zika virus: Inflammation and behavioral sequela in mice. *PLoS Pathog.* 16:e1008689. 10.1371/journal.ppat.1008689 33301527 PMC7728251

[B18] Kolb B. Gibb R. (2011). Brain plasticity and behaviour in the developing brain. *J. Can. Acad. Child Adolesc. Psychiatry* 20 265–276. 22114608 PMC3222570

[B19] Lemons M. L. Sandy J. D. Anderson D. K. Howland D. R. (2001). Intact aggrecan and fragments generated by both aggrecanse and metalloproteinase-like activities are present in the developing and adult rat spinal cord and their relative abundance is altered by injury. *J. Neurosci.* 21 4772–4781. 10.1523/JNEUROSCI.21-13-04772.2001 11425904 PMC6762363

[B20] Luo Q. S. Luo H. C. Chen X. P. Yan P. Fu H. D. Huang H. N. (2018). The expression of MMP19 and its clinical significance in glioma. *Int. J. Clin. Exp. Pathol.* 11 5407–5412. 31949623 PMC6963022

[B21] Manangeeswaran M. Ireland D. D. C. Verthelyi D. (2016). Zika (PRVABC59) infection is associated with T cell Infiltration and neurodegeneration in CNS of immunocompetent neonatal C57Bl/6 mice. *PLoS Pathog.* 12:e1006004. 10.1371/journal.ppat.1006004 27855206 PMC5113993

[B22] Mao M. J. Yu H. L. Wen Y. Z. Sun X. Y. Xu C. Y. Gao Y. Z. (2022). Deficit of perineuronal net induced by maternal immune activation mediates the cognitive impairment in offspring during adolescence. *Behav. Brain Res.* 434:114027. 10.1016/j.bbr.2022.114027 35905839

[B23] Matthews R. T. Kelly G. M. Zerillo C. A. Gray G. Tiemeyer M. Hockfield S. (2002). Aggrecan glycoforms contribute to the molecular heterogeneity of perineuronal nets. *J. Neurosci.* 22 7536–7547. 10.1523/JNEUROSCI.22-17-07536.2002 12196577 PMC6757962

[B24] Mauney S. A. Athanas K. M. Pantazopoulos H. Shaskan N. Passeri E. Berretta S. (2013). Developmental pattern of perineuronal nets in the human prefrontal cortex and their deficit in schizophrenia. *Biol. Psychiatry* 74 427–435. 23790226 10.1016/j.biopsych.2013.05.007 PMC3752333

[B25] Milev P. Maurel P. Chiba A. Mevissen M. Popp S. Yamaguchi Y. (1998). Differential regulation of expression of hyaluronan-binding proteoglycans in developing brain: Aggrecan, versican, neurocan, and brevican. *Biochem. Biophys. Res. Commun.* 247 207–212. 10.1006/bbrc.1998.8759 9642104

[B26] Mirzadeh Z. Alonge K. M. Cabrales E. Herranz-Perez V. Scarlett J. M. Brown J. M. (2019). Perineuronal net formation during the critical period for neuronal maturation in the hypothalamic arcuate nucleus. *Nat. Metab.* 1 212–221. 10.1038/s42255-018-0029-0 31245789 PMC6594569

[B27] Miyata S. Kitagawa H. (2016). Chondroitin 6-sulfation regulates perineuronal net formation by controlling the stability of aggrecan. *Neural Plast.* 2016:1305801. 10.1155/2016/1305801 27057358 PMC4738747

[B28] Muri L. Leppert D. Grandgirard D. Leib S. L. (2019). MMPs and ADAMs in neurological infectious diseases and multiple sclerosis. *Cell. Mol. Life Sci.* 76 3097–3116. 10.1007/s00018-019-03174-6 31172218 PMC7079810

[B29] Paylor J. W. Lins B. R. Greba Q. Moen N. De Moraes R. S. Howland J. G. (2016). Developmental disruption of perineuronal nets in the medial prefrontal cortex after maternal immune activation. *Sci. Rep.* 6:37580. 10.1038/srep37580 27876866 PMC5120325

[B30] Poretz R. D. Tang M. Vucenik I. (1986). The separation of lymphocyte subpopulations with lectins. *Immunol. Invest.* 15 521–529. 3793157 10.3109/08820138609026692

[B31] Reinhard S. M. Razak K. Ethell I. M. (2015). A delicate balance: Role of MMP-9 in brain development and pathophysiology of neurodevelopmental disorders. *Front. Cell. Neurosci.* 9:280. 10.3389/fncel.2015.00280 26283917 PMC4518323

[B32] Roughley P. J. Mort J. S. (2014). The role of aggrecan in normal and osteoarthritic cartilage. *J. Exp. Orthop.* 1:8. 10.1186/s40634-014-0008-7 PMC4648834 26914753

[B33] Schindelin J. Arganda-Carreras I. Frise E. Kaynig V. Longair M. Pietzsch T. (2012). Fiji: An open-source platform for biological-image analysis. *Nat. Methods* 9 676–682. 10.1038/nmeth.2019 22743772 PMC3855844

[B34] Seeger G. Brauer K. Hartig W. Bruckner G. (1994). Mapping of perineuronal nets in the rat brain stained by colloidal iron hydroxide histochemistry and lectin cytochemistry. *Neuroscience* 58 371–388. 10.1016/0306-4522(94)90044-2 7512240

[B35] Shi W. Wei X. Wang X. Du S. Liu W. Song J. (2019). Perineuronal nets protect long-term memory by limiting activity-dependent inhibition from parvalbumin interneurons. *Proc. Natl. Acad. Sci. U.S.A.* 116 27063–27073. 10.1073/pnas.1902680116 31843906 PMC6936502

[B36] Sigal Y. M. Bae H. Bogart L. J. Hensch T. K. Zhuang X. W. (2019). Structural maturation of cortical perineuronal nets and their perforating synapses revealed by superresolution imaging. *Proc. Natl. Acad. Sci. U.S.A.* 116 7071–7076. 10.1073/pnas.1817222116 30890637 PMC6452715

[B37] Slaker M. L. Harkness J. H. Sorg B. A. (2016). A standardized and automated method of perineuronal net analysis using Wisteria floribunda agglutinin staining intensity. *IBRO Rep.* 1 54–60. 10.1016/j.ibror.2016.10.001 28713865 PMC5507617

[B38] Sonntag M. Blosa M. Schmidt S. Reimann K. Blum K. Eckrich T. (2018). Synaptic coupling of inner ear sensory cells is controlled by brevican-based extracellular matrix baskets resembling perineuronal nets. *BMC Biol.* 16:99. 10.1186/s12915-018-0566-8 30253762 PMC6156866

[B39] Sorg B. A. Berretta S. Blacktop J. M. Fawcett J. W. Kitagawa H. Kwok J. C. (2016). Casting a wide net: Role of perineuronal nets in neural plasticity. *J. Neurosci.* 36 11459–11468. 10.1523/JNEUROSCI.2351-16.2016 27911749 PMC5125213

[B40] Tewari B. P. Chaunsali L. Campbell S. L. Patel D. C. Goode A. E. Sontheimer H. (2018). Perineuronal nets decrease membrane capacitance of peritumoral fast spiking interneurons in a model of epilepsy. *Nat. Commun.* 9:4724. 10.1038/s41467-018-07113-0 30413686 PMC6226462

[B41] Tewari B. P. Chaunsali L. Prim C. E. Sontheimer H. (2022). A glial perspective on the extracellular matrix and perineuronal net remodeling in the central nervous system. *Front. Cell. Neurosci.* 16:1022754. 10.3389/fncel.2022.1022754 36339816 PMC9630365

[B42] Ueno H. Fujii K. Suemitsu S. Murakami S. Kitamura N. Wani K. (2018a). Expression of aggrecan components in perineuronal nets in the mouse cerebral cortex. *IBRO Rep.* 4 22–37. 30135949 10.1016/j.ibror.2018.01.002 PMC6084874

[B43] Ueno H. Suemitsu S. Murakami S. Kitamura N. Wani K. Matsumoto Y. (2018b). Juvenile stress induces behavioral change and affects perineuronal net formation in juvenile mice. *BMC Neurosci.* 19:41. 10.1186/s12868-018-0442-z 30012101 PMC6048828

[B44] van’t Spijker H. M. Kwok J. C. F. (2017). A sweet talk: The molecular systems of perineuronal nets in controlling neuronal communication. *Front. Integr. Neurosci.* 11:33. 10.3389/fnint.2017.00033 29249944 PMC5717013

[B45] Venturino A. Schulz R. De Jesus-Cortes H. Maes M. E. Nagy B. Reilly-Andujar F. (2021). Microglia enable mature perineuronal nets disassembly upon anesthetic ketamine exposure or 60-Hz light entrainment in the healthy brain. *Cell Rep.* 36:109313. 10.1016/j.celrep.2021.109313 34233180 PMC8284881

[B46] Wegrzyn D. Manitz M. P. Kostka M. Freund N. Juckel G. Faissner A. (2021). Poly I:C-induced maternal immune challenge reduces perineuronal net area and raises spontaneous network activity of hippocampal neurons in vitro. *Eur. J. Neurosci.* 53 3920–3941. 10.1111/ejn.14934 32757397

[B47] Wingert J. C. Sorg B. A. (2021). Impact of perineuronal nets on electrophysiology of parvalbumin interneurons, principal neurons, and brain oscillations: A review. *Front. Synaptic Neurosci.* 13:673210. 10.3389/fnsyn.2021.673210 34040511 PMC8141737

