# Supplementary figures and images for: Neonatal Zika virus infection causes transient perineuronal net degradation
Source: Front Cell Neurosci. 2023 Jul 11;17:1187425. doi: 10.3389/fncel.2023.1187425 (PMC10366369; doi:10.3389/fncel.2023.1187425)

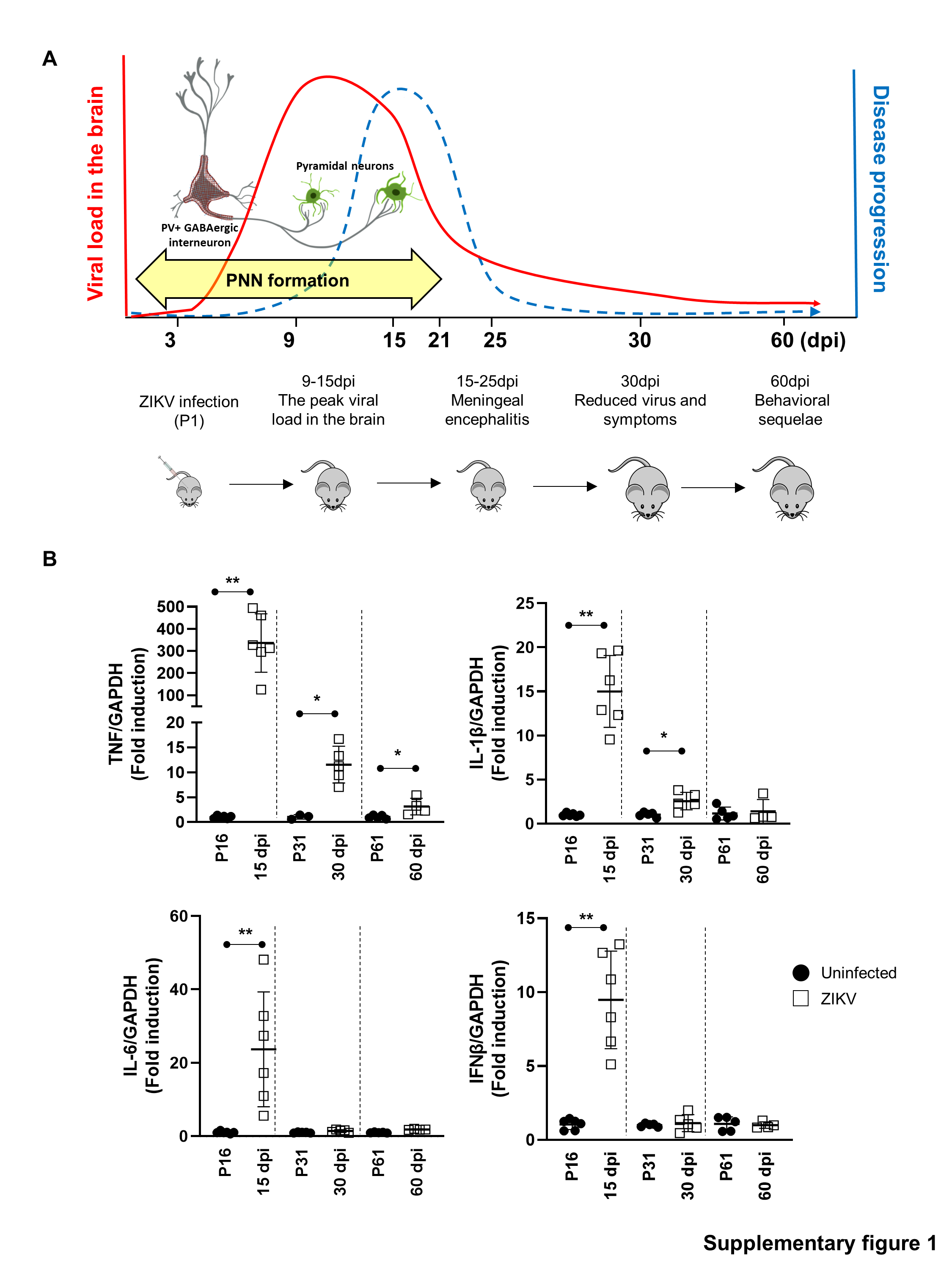

Supplement: Supplementary Figure 1 — The disease and inflammation progression during ZIKV infection. (A) Schematic diagram depicting the disease progression of ZIKV infection. P1 C57BL/6 mice infected with ZIKV show the CNS infection that peaks at 9–15 dpi, together with a transient meningeal encephalitis characterized by unsteady gait, kinetic tremors, ataxia and seizures that appear 10–12 dpi and subside by 26–30 dpi. ZIKV-infected mice can show behavioral sequelae as late as 60 dpi. (B) Relative mRNA levels of TNF, IL-1β, IL-6, and IFNβ were determined in the brain of uninfected or ZIKV-infected mice at 15, 30, and 60 dpi using real-time RT PCR. Data shown as means ± S.D. (n = 4–6 mice for each group). *P < 0.05, **P < 0.01 (student’s t-test). [file Image_1.TIF]

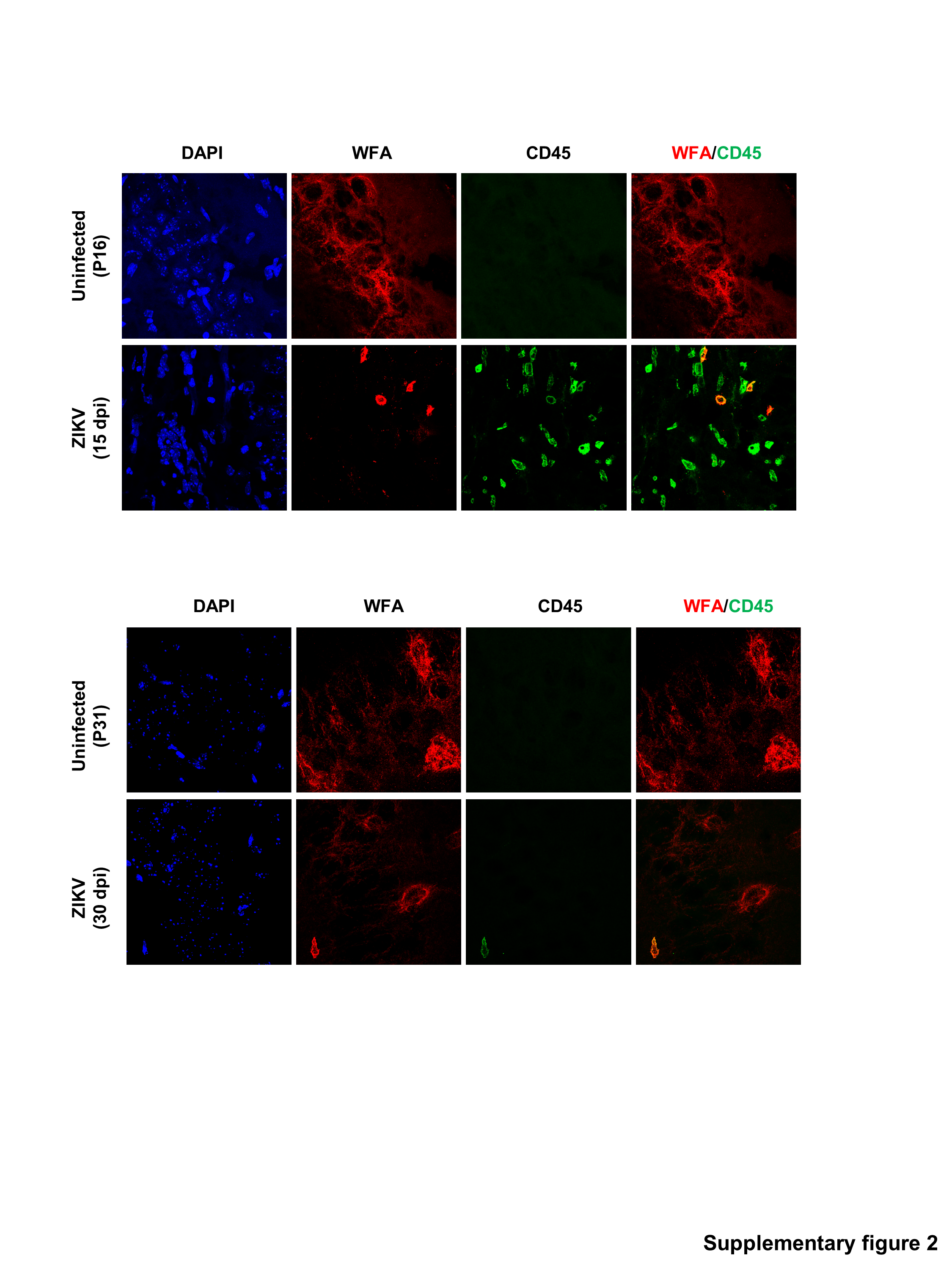

Supplement: Supplementary Figure 2 — Wisteria floribunda agglutinin (WFA) staining is colocalized with CD45 staining in round shaped cells observed in ZIKV-infected brains. Confocal imaging of WFA and CD45 staining in the CA2 regions of the brain from uninfected or ZIKV-infected mice at P16/15 dpi and P31/30 dpi. The images show representative immunofluorescence staining for DAPI (blue), WFA (red) and CD45 (green) in brain sections. [file Image_2.TIF]

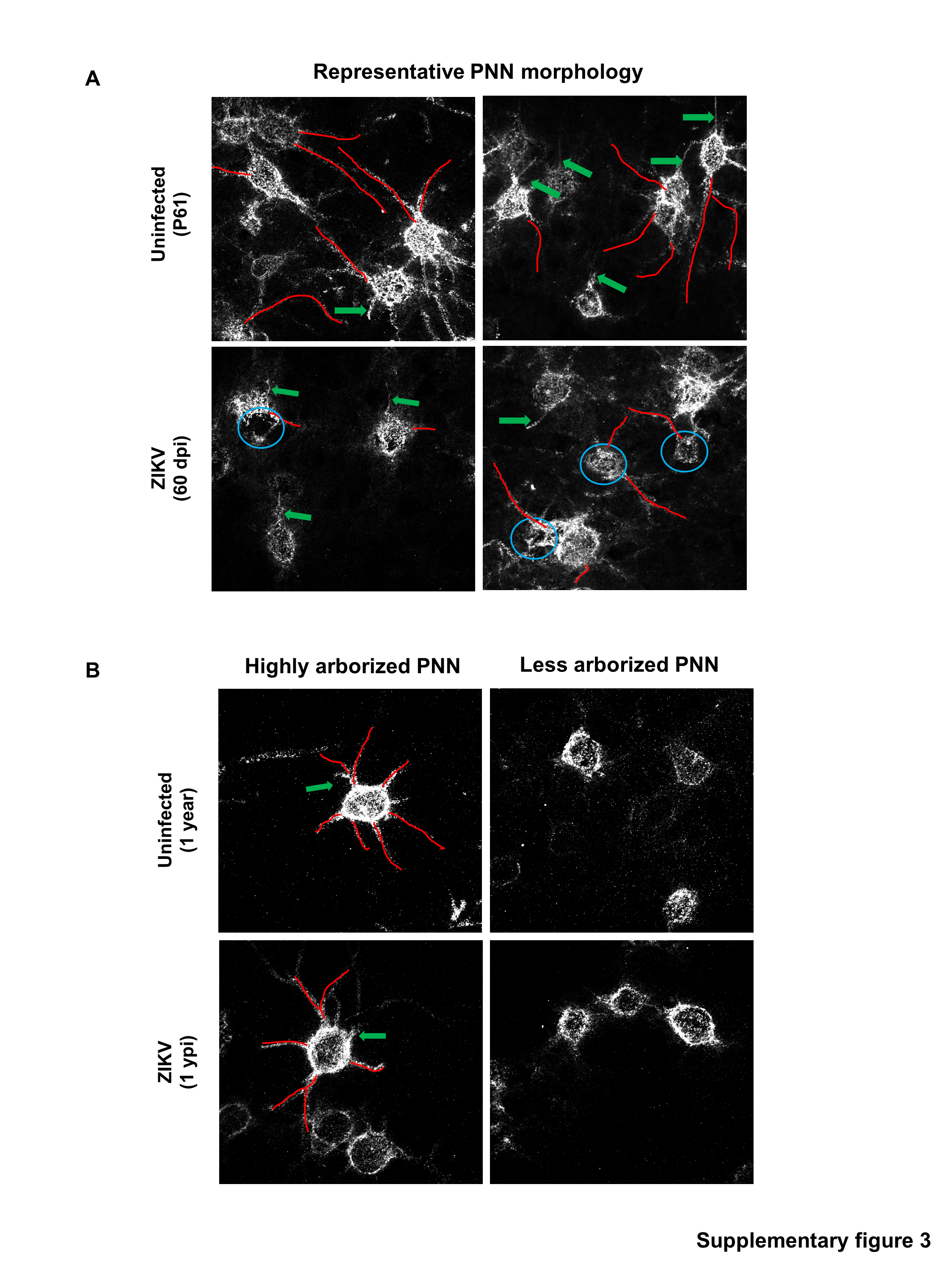

Supplement: Supplementary Figure 3 — Perineuronal net morphology in control and convalescent mice. Two representative confocal images (63×) show immunofluorescence staining for WFA in SSC regions of age-matched controls and convalescent mice at 60 dpi (A) and 1 ypi (B). Blue circles, ghost PNNs; red lines, PNN on dendritic arbor; green arrows, PNN on AIS. [file Image_3.TIF]

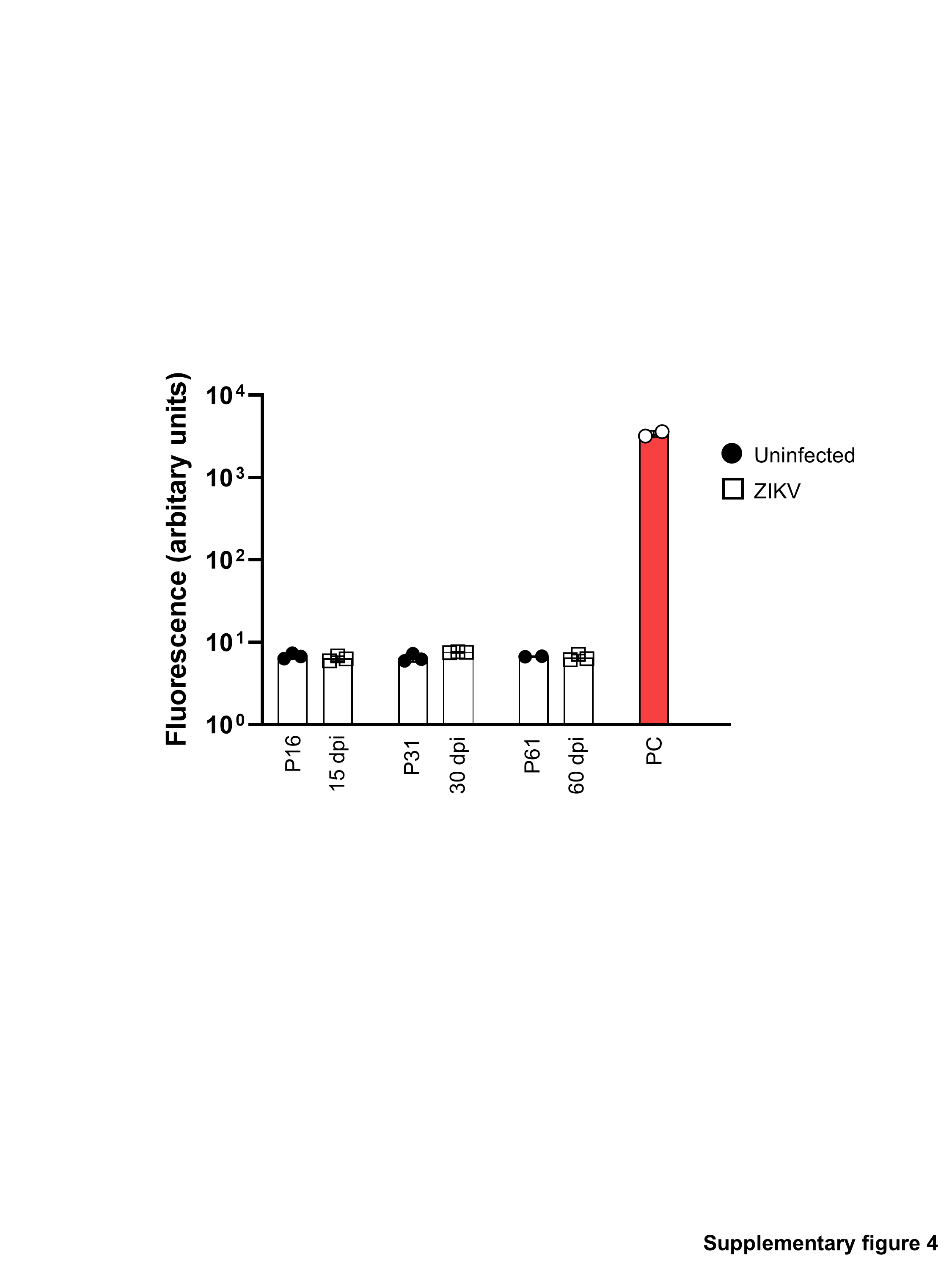

Supplement: Supplementary Figure 4 — Gelatinase/collagenase activity in the brain of ZIKV-infected mice. Gelatinase/collagenase activity was measured in the brain lysates from uninfected and ZIKV-infected mice at the indicated time point. PC, positive control (0.1 U/mL of Clostridium collagenase) is red bar. n = 2–3 mice for each group. [file Image_4.TIF]

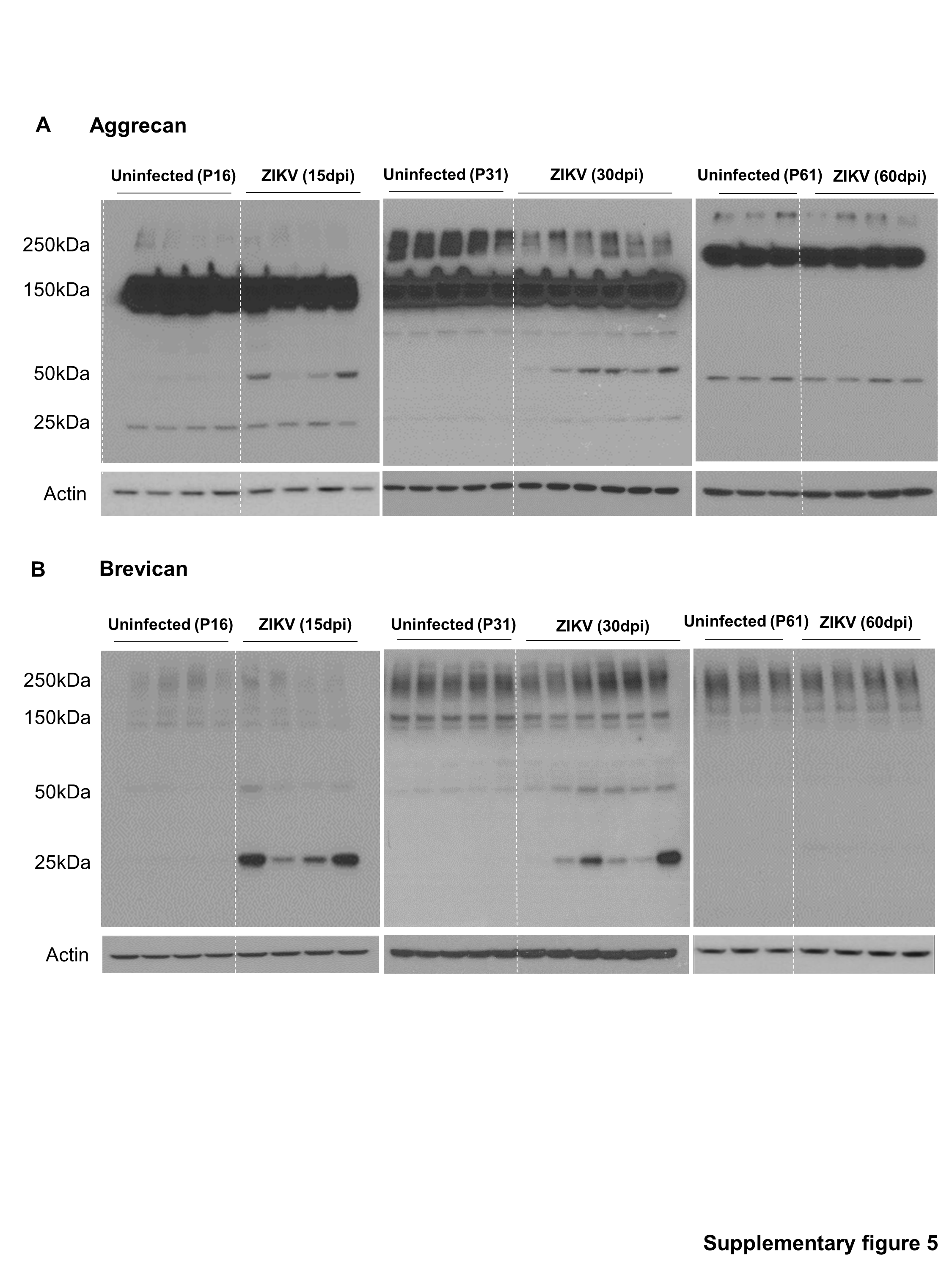

Supplement: Supplementary Figure 5 — Uncropped gel images. Full length gel images of aggrecan (A) and brevican (B) corresponding to the cropped images shown in Figures 1E, H, 2E, H, 3E, H. [file Image_5.TIF]
